# Innovative Approaches to Depression Therapy: The Antidepressant Efficacy and Mechanisms of General Anesthetics

**DOI:** 10.2174/011570159X458197260226055817

**Published:** 2026-04-16

**Authors:** Xiangying Wei, Shan Xu, Zhaoqiong Zhu

**Affiliations:** 1 Department of Anesthesiology, Affiliated Hospital of Zunyi Medical University, Zunyi, 563003, China;; 2 Early Clinical Research Ward, Affiliated Hospital of Zunyi Medical University, Zunyi, 563003, China

**Keywords:** Depression, general anesthetics, anesthetic therapeutics, antidepressant, treatment-resistant depression (TRD), Ketamine

## Abstract

Depression represents a critical global health burden, ranking as the second leading cause of disability worldwide. Although drug therapies remain an important cornerstone in treating depression, we must acknowledge their significant shortcomings: they often take too long to work, cause difficult side effects, and fail to prevent relapse. Critically, while current drugs target specific symptoms, the disorder itself stems from a far more complex interplay of genetic, environmental, and biological factors. Notably, emerging evidence indicates that several general anesthetics exhibit both rapid and sustained antidepressant effects. Ketamine, through blocking N-methyl-D-aspartate (NMDA) receptors, has established a new paradigm for rapid-acting antidepressant intervention. Building on this breakthrough, researchers are now finding that other agents, including propofol, isoflurane, and specific opioids, show promising results across preclinical and clinical settings. This expanding evidence base significantly advances the therapeutic scope of anesthesiology while addressing treatment-resistant depression (TRD) affecting approximately one-third of patients. This review examines intravenous/inhalational anesthetics and opioid analgesics for depression management, synthesizing contemporary preclinical and clinical evidence with particular focus on randomized controlled trials and mechanistic studies. Our review highlights the specific biological changes that drive these effects. Beyond monoamine regulation, key factors include modulation of glutamate and GABA signals, which contribute to the restoration of synaptic plasticity in damaged brain circuits. Through combining what is known with what remains unclear, specifically focusing on dosing and long-term outcomes. We argue that targeting these mechanisms is key to creating new rapid antidepressants for patients who do not respond to standard care. Yet, success depends on balancing benefits against risks and establishing consistent treatment rules.

## 1 INTRODUCTION

Depression is more than just a fleeting mood; it is a widespread clinical disorder defined by persistent sadness and a profound loss of interest in daily life. Approximately 280 million individuals globally experience depression, and it is anticipated that by 2030, it will become the predominant disease burden worldwide [1]. Managing depression typically requires a multimodal approach that combines psychological and physical support. Despite this variety, drug therapy continues to serve as the primary foundation for most treatment protocols. While antidepressants help to alleviate depression symptoms to some degree, they include limitations like delayed onset of action, ease of tolerance, and numerous side effects. Approximately 30% of individuals have inadequate responses to conventional antidepressant pharmacology [2]. Trivedi *et al*. [3] conducted a 14-week surveillance of 2,876 outpatients with Major Depressive Disorder (MDD), revealing that roughly 28% of patients did not exhibit symptom improvement until they had received standard antidepressant treatment for over 8 weeks. Blackburn TP [4] research indicates that the effectiveness of prolonged antidepressant use diminishes over time and may be associated with medication resistance. Common side effects of antidepressants encompass cardiovascular complications, including arrhythmia and hypertension, with an elevated risk in elderly individuals [5]. Furthermore, numerous antidepressants induce metabolic syndrome, and an analysis of National Health and Nutrition Examination Survey (NHANES) data from 2005 to 2018 revealed that antidepressant medication use was associated with a 24% increased odds of metabolic syndrome compared to non-users. Notably, participants prescribed tricyclic antidepressants (TCAs) exhibited a substantially elevated risk, demonstrating a 127% higher odds of metabolic syndrome relative to non-medication users [6]. A randomized double-blind controlled trial conducted by K. El Asmar *et al*. [7] revealed that 17.2% of patients experienced a 5% weight increase after 30 days of conventional antidepressant therapy, and the incidence of metabolic syndrome was approximately 8.6 times greater in the responder group compared to the non-responder group. This not only impacts the patient's psychological condition but may also be attributable to obesity-related health issues. Moreover, an analysis of data from the European Drug Monitoring Program (AMSP) spanning 1993 to 2014 indicated 83 instances of suicidal adverse reactions linked to antidepressant medications, with Selective Serotonin Reuptake Inhibitors (SSRIs) being the predominant cause of these suicidal adverse events, accounting for 57% of cases [8]. This indicates that current antidepressant medications possess significant potential for enhancement, and the pursuit of novel therapeutic approaches and pharmacological action targets remains a primary emphasis in depression research.

General anesthetics are a class of drugs that inhibit central nervous system activity, resulting in a transient loss of awareness, sensation, and reflexes. In 2000, Yale University's Berman [9]. Professor's research team demonstrated for the first time that low-dose ketamine can yield immediate and effective antidepressant benefits, particularly in patients with TRD. The findings in this paper represents the most significant breakthrough in psychiatry in nearly half a century. Furthermore, it highlights the promising potential of general anesthetics in antidepressant therapy. As research progresses, scientific studies consistently affirm the efficacy and benefits of ketamine as an antidepressant. Simultaneously, numerous findings indicate that dexmedetomidine, sevoflurane, narcotic analgesics, and other general anesthetics have been effective in mitigating depressed symptoms [10, 11]. These discoveries offer novel insights and methodologies for the treatment of depression.

## 2 INTRAVENOUS ANESTHETICS

### 2.1 Ketamine

Ketamine induces potent analgesia, sedation, and amnesia, manifesting as a unique state of ‘dissociative anesthesia.’ Mechanistically, it exerts general anesthetic effects by antagonizing excitatory NMDA receptors [12]. In 2000, Berman employed a placebo-controlled, double-blind methodology to randomize 9 patients with MDD (2 of whom withdrew during the study). Participants received either saline or 0.5 mg/kg ketamine (subanesthetic dose), with the Hamilton Depression Rating Scale (HDRS) assessed at 80 minutes, 230 minutes, 24 hours, 48 hours, and 72 hours post-infusion, along with the Beck Depression Inventory (BDI) and additional depression scale evaluations. The findings indicated that the HRDS scores of individuals with depression diminished more markedly within 72 hours post-ketamine infusion, implying that subanesthetic dose ketamine can swiftly mitigate the depressive symptoms in patients with major depression [9]; This finding triggered decades of investigation into whether general anesthetics could treat mental health conditions. Beyond psychiatry, it also reshaped how we approach therapeutic anesthesia. In 2006, the National Institute of Mental Health (NIMH) executed a randomized controlled trial involving 18 patients with treatment-resistant depression. Evidence demonstrates that a single sub-anesthetic dose of ketamine produces rapid and significant antidepressant effects in patients with treatment-resistant depression. Notably, this therapeutic benefit persists for 1 week [13]. Subsequent research has investigated whether repeated infusions of ketamine, administered twice [14] or thrice [15], sustain their effects in depressed patients. Findings indicate that repeated ketamine administration moderately extends the antidepressant effect; however, relapse remains a possibility 18-19 days post-infusion cessation [16, 17]. J.L. Phillips *et al*. [18] conducted a maintenance phase study involving 41 patients with treatment-resistant depression, who received three weekly infusions of ketamine, followed by four weeks of continuous intravenous infusion at a dosage of 0.5 mg/kg weekly. The results indicated that MADRS scores remained largely unchanged, suggesting that maintenance infusions of ketamine are essential for sustained antidepressant effects; however, additional research is needed to assess its safety and tolerability. Finding the right dose of ketamine for depression remains a central challenge. Researchers have extensively debated how much is needed to balance efficacy with safety. In a four-week randomized, double-blind, controlled trial, the Loo research group [19] found that ketamine demonstrated superior antidepressant efficacy in the flexible dosage group (0.5-0.9 mg/kg) compared with the fixed dosage group (0.5 mg/kg), suggesting that individualized dosing is more effective. Conversely, a randomized controlled experiment by Glue *et al*. [20] revealed no significant difference in the HADS-Depression and Anxiety Scale between patients in the 0.5 mg dose group and those in the 1 mg/kg dose group. The antidepressant benefits of the 1 mg/kg dose were maintained longer, resulting in decreased depression and anxiety scores in patients within 1 hour of ketamine administration, and lasted for up to 7 days. The quantitative effects of ketamine as an antidepressant are not entirely positively correlated. Zanos *et al*. [21] demonstrated that by administering varying doses (1, 3, 10, 30, and 100 mg/kg) of ketamine to mice, its efficacy in reversing Learned Helplessness (LH) was evaluated after 24 hours. At a dosage of 10 mg/kg, ketamine markedly diminished the incidence of escape failures in vulnerable mice. A tendency to understate escape failures was observed at a dosage of 30 mg/kg. The antidepressant-like effect of ketamine was entirely abolished at a dosage of 100 mg/kg. These findings point to a critical therapeutic window: while low, sub-anesthetic doses often produce lasting relief, higher amounts appear to be counterproductive, potentially blocking these very benefits. Therefore, the optimal dosage and administration methods warrant further exploration. Recently, scientists have focused on esketamine (the S-enantiomer), a version that binds more tightly to target receptors. Because it offers potent relief with fewer neurological risks, it has become a preferred option over standard ketamine. In 2019, the U.S. approved the nasal spray formulation of the medication. The FDA will be utilized in the clinical management of depression.

The antidepressant effects of ketamine are realized through various interconnected processes:

#### 2.1.1 Mechanisms of Neurotransmitter Systems

The "monoamine hypothesis" remains the classic theoretical framework for depression, guiding traditional antidepressants to elevate CNS monoamine levels. Consequently, ketamine’s potential modulation of these neurotransmitters has garnered significant scientific interest. A study by Y. Ago *et al*. [22] examined the effects of ketamine and its metabolites on monoamine neurotransmitters in the prefrontal cortex (PFC) using microdialysis. The results indicated that ketamine influenced 5-hydroxytryptamine (5-HT), dopamine (DA), and norepinephrine (NE) with an extended duration of action; however, the relationships between their quantitative and temporal effects were not entirely consistent, necessitating more precise delineation for further clarification. A recent animal study administered saline or ketamine (10 mg/kg) injections to mice and used two-photon glutamate release/imaging to assess dendritic spine development in medial prefrontal cortex (mPFC) neurons at various time points (2 to 72 hours). Glutamate-induced dendritic spiking was considerably augmented from 2 to 4 hours post-ketamine administration, confirming that ketamine swiftly boosts glutamate-induced dendritic spiking in mPFC pyramidal neurons, resulting in a quick antidepressant effect [23]. Historically, NMDA receptor antagonism was not considered the primary mechanism underlying ketamine’s antidepressant effects [12, 24]. Subsequent study revealed that ketamine may alleviate depression by targeting NMDA receptors in particular regions of the brain. The lateral habenula (LHb) functions as the "anti-reward center" in the brain, with its clustered firing pattern reliant on NMDA receptors, the target proteins of ketamine. Hailan Hu's team [25] administered ketamine into the bilateral LHb nuclei of congenitally learned helpless (cLH) rats. One hour post-administration, ketamine significantly reduced immobility in the Forced Swim Test (FST) and increased sucrose intake in the Sucrose Preference Test (SPT). These results confirm that ketamine exerts rapid antidepressant effects by terminating NMDAR-dependent burst firing. Furthermore, a single systemic injection was found to block this bursting activity in the LHb for up to 24 hours. This effectively disinhibits downstream dopaminergic and serotonergic neurons, thereby rapidly ameliorating depressive symptoms [26].

#### 2.1.2 Neurotrophic Factor Signaling Pathway

α-amino-3-hydroxy-5-methyl-4-isoxazolepropionic acid (AMPA) receptors and Brain-Derived Neurotrophic Factor (BDNF) are critical for neurogenesis, dendritic spine development, and the modulation of synaptic transmission [27]. Li *et al*. [28] administered a single intraperitoneal injection of ketamine (10 mg/kg) to mice predisposed to postoperative depression; behavioral evaluations conducted 30 minutes later revealed a significant reduction in BDNF-TrkB signaling within the mPFC and hippocampus of POD-susceptible mice compared to the control group. Following ketamine administration, aberrant levels of BDNF and the TrkB receptor were normalized, indicating that ketamine exerts a notable ameliorative effect on postoperative depression-like symptoms by modulating the BDNF-TrkB signaling pathway. AAV-mediated selective knockout of BDNF or TrkB was performed in the CA1 or CA3 regions of adult mice. Three weeks post-surgery, field excitatory postsynaptic potentials (fEPSPs) recordings revealed that the typical synaptic potentiation induced by ketamine (20 μM) was absent in CA3-CA1 synapses. This demonstrates that disrupting BDNF or TrkB blocks ketamine-induced, AMPA receptor-mediated plasticity, underscoring the critical role of the BDNF-TrkB pathway in its antidepressant mechanism [29].

#### 2.1.3 Cytokines and Neuroinflammatory Pathways

Chronic stress triggers peripheral and central inflammation, a process implicated in the pathogenesis of depression. Based on this theory, animal models such as corticosterone-induced depression and Chronic Unpredictable Mild Stress (CUMS) have been successfully established. In clinical studies, a subanesthetic dose of ketamine was found to reduce depression scale scores and TNF-α levels in patients with TRD, and there was a correlation between the two reductions [30]. Mice subjected to CUMS exhibited elevated pro-inflammatory cytokines (IL-1β, TNF-α, IL-6), increased reactive microglia, and upregulated hippocampal TLR4/p38 and P2X7 receptors. Notably, ketamine treatment effectively reversed these biochemical abnormalities. It is suggested that the antidepressant effect of ketamine in mice is related to its down-regulation of pro-inflammatory factors [31]. In a systematic evaluation, the authors analyzed 807 studies evaluating the effects of ketamine on inflammatory markers in patients with monophasic and biphasic depression as well as in animal models of depression, and found that the animal studies provided strong support for ketamine-induced reductions in pro-inflammatory cytokines [32]. mTOR is a classical serine/acid kinase that regulates cell growth, metabolism. E. Garro-Martínez *et al*. [33] found that knockdown of mTOR induced a depressive-like state in mice. Reports indicate that ketamine rapidly enhances mTOR phosphorylation and its downstream targets, initiating signaling cascades that drive its antidepressant effects [34, 35]. In a randomized study, 20 MDD patients received oral rapamycin (6 mg), an mTORC1 inhibitor, or placebo two hours prior to an intravenous ketamine infusion (0.5 mg/kg over 40 min). Serial MADRS assessments revealed that rapamycin pretreatment prolonged ketamine’s antidepressant effects. Notably, at two weeks, the rapamycin group exhibited significantly higher response (41%) and remission (29%) rates compared to placebo, further substantiating the critical role of mTOR signaling in ketamine’s antidepressant efficacy [36].

#### 2.1.4 Pathway of Oxidative Stress Response

Oxidative stress arises when an imbalance between excessive oxidation and insufficient antioxidant defense leads to free radical accumulation, disrupting cellular homeostasis. Consistently, MDD patients exhibit elevated plasma and serum oxidative biomarkers (MDA, 8-OHdG, F2-isoprostanes) accompanied by reduced antioxidant levels (*e.g*., SOD) [37]. Integrated metabolomic and proteomic profiling of the ketamine-treated mouse hippocampus identified mitochondrial energy metabolism and antioxidant defense systems as downstream effectors of the drug’s response [38]. A study investigating the impact of a single ketamine dose on depression-related behaviors and oxidative stress in adult rats subjected to maternal deprivation revealed that ketamine mitigates depression by inhibiting oxidative stress [39], a finding corroborated by Carlessi *et al* [40]. Nonetheless, there may be gender disparities in the mechanism by which ketamine operates through this pathway [40, 41].

Ketamine, as a novel antidepressant, has substantial therapeutic potential due to its synergistic effects across multiple pathways, whether administered by intravenous injection or intranasal spray. Nonetheless, ketamine is associated with dissociative hallucinations, addiction, and other side effects that warrant attention [42, 43]; prolonged usage may lead to dependence. Consequently, while the effectiveness of ketamine as a general anesthetic in antidepressant therapy is evident, its safety must also be considered. To unlock the next generation of safer, more potent antidepressants, we must first pinpoint exactly how ketamine works in the brain. Identifying its specific targets is key to mitigating its risks while retaining its benefits.

### 2.2 Dexmedetomidine

Dexmedetomidine (DEX) is a highly selective agonist of the α2-adrenergic receptor (α2-AR) that induces sedative, anxiolytic, and analgesic effects through the activation of α2-AR in the central nervous system [44]. α2-ARs are primarily located on the presynaptic membranes of adrenergic neurons in the CNS. Upon activation, they inhibit norepinephrine release *via* a negative feedback mechanism. Shams [45] Randomized depressed patients undergoing electroconvulsive treatment (ECT) were allocated into two groups. Patients administered DEX + chlorophenol (a combination anesthetic of ketamine and propofol) exhibited a more pronounced antidepressant effect. They exhibited greater hemodynamic stability and a lower incidence of agitation compared to the chlorophenol group. A study conducted by Y. Liu *et al*. [46] randomized patients with TRD into two cohorts: one receiving ECT and the other undergoing ten DEX treatments. Utilizing the 24-item Hamilton Depression Rating Scale (HDRS-24), Results indicate that depression severity was reduced to mild levels after just two DEX infusions, whereas at least six ECT sessions were required to achieve a comparable outcome. A meta-analysis encompassing 13 randomized controlled trials with a total of 1,711 participants revealed that DEX administered during the early postpartum period significantly mitigated postpartum depression (PPD), enhanced postoperative analgesia and sleep quality, and offered the additional benefit of reducing adverse effects such as maternal nausea, vomiting, and chills [47]. We suggest that DEX acts on multiple fronts to combat depression. It appears to rebalance neurotransmitters and dampen harmful inflammation, while simultaneously boosting the production of BDNF. Zhou [48], in a randomized controlled trial examining prenatal and postnatal plasma BDNF levels in individuals with depression, it was observed that patients administered DEX during the early postnatal phase exhibited elevated levels of BDNF and pro-BDNF, with the alterations in pro-BDNF being particularly significant. This implies that DEX may exert an antidepressant effect by modulating BDNF levels *in vivo*. In a murine model of sleep loss-induced depressed behavior, Moon *et al*. [49] discovered that DEX mitigated depressive symptoms resulting from sleep deprivation within a brief duration (6 days). The method included DEX enhancing 5-HT synthesis in the Dorsal Raphe Nucleus (DRN), reducing DA levels in the nucleus accumbens(NAc), and up-regulating D1 DA receptor levels in mood-related brain areas. In a murine model of chronic pain-induced depression, researchers discovered that DEX effectively mitigated the depressed phenotype by facilitating hippocampal neurogenesis [50]. While evidence suggests DEX holds antidepressant potential, further preclinical and clinical studies are required to fully validate its efficacy and safety profile.

### 2.3 Propofol

 Propofol induces sleep by amplifying the brain’s natural inhibitory system. Specifically, it targets GABA receptors to drive chloride into cells; this effectively ‘quiets’ neuronal activity (hyperpolarization), resulting in deep sedation. Besides its widespread use in general anesthesia, it is the most commonly used sedative in ECT therapy. Zeng *et al*. [51] evaluated the effectiveness of ketamine-ECT against propofol-ECT in treating individuals with MDD. The study revealed that the level of depression remission in the propofol group was comparable to that in the ketamine group, indicating a certain efficacy of propofol as an antidepressant. An open-label trial administered 10 infusions of high-dose propofol (9-20 mg/kg) three times weekly demonstrated a mean reduction of 20 points (range 0-45) in total HDRS scores post-treatment, with 6 out of 10 subjects achieving positive treatment response criteria (>50% improvement) and five meeting remission criteria (HDRS-24<10) [52], This suggests that high-dose propofol may trigger rapid and sustained antidepressant effects by inducing burst suppression. This EEG-defined state of neural hyperexcitation resembles ECT but entails fewer adverse events [52]. Current limited research suggests propofol targets the DA transporter to upregulate NAc DA in chronic stress models, thereby alleviating anhedonia with a low risk of addiction [53]. We propose that propofol triggers euphoria by engaging this reward circuit. This surge in positive mood likely drives its ability to lift depression so quickly. A recent study by Wang *et al*. [54] showed that propofol activates hippocampal CaMKIIα signaling to upregulate PSD-95 and GluA1. Crucially, the treatment rescues synaptic plasticity. This cellular repair drives a clear reversal of depressive behaviors in the mice, providing robust support for our initial theory. Nonetheless, certain investigations have proposed a contrary perspective. Song *et al*. [55] administered a single intraperitoneal dose of propofol (75 mg/kg) to 8-10 week-old mice exhibiting depressive behavior, resulting in a significant reduction in struggle time during the tail-hanging, forced swim, and absent-field tests, indicating that a single dose of propofol may lack enduring antidepressant-like effects. A study conducted by Daniel *et al*. [56] on the effects of FST on mouse behavior 24 hours post a single intraperitoneal administration of 50 mg/kg of propofol yielded a consistent finding. Therefore, conclusions regarding the antidepressant efficacy of propofol remain inconsistent. Specific dosing studies are currently lacking. Future research must further elucidate the underlying mechanisms and optimize administration strategies.

### 2.4 Benzodiazepines

 Benzodiazepines (BZDs) exert sedative and hypnotic effects by enhancing the activity of GABA. The findings of BZDs in the treatment of depression have been inconsistent. Reports up to May 2019 indicate that during the early phase (0-4 weeks), combining benzodiazepines with antidepressants is more effective than antidepressant monotherapy in improving symptoms and response rates. However, this advantage ceases to exist at 5-12 weeks or beyond [57]. In animal studies, intraperitoneal midazolam ameliorated depressive-like behaviors in rats exhibiting behavioral despair. ELISA analysis revealed elevated allopregnanolone levels in the PFC and hippocampus (HC). Notably, this elevation was reversed by the TSPO antagonist PK11195, suggesting that the antidepressant mechanism is linked to TSPO [58]. In contrast, a prospective study indicated that TRD patients with long-term BZD use exhibited more severe depressive symptoms and functional impairment than controls. Extending treatment failed to boost one-year remission rates. Paradoxically, longer exposure was linked to worse outcomes, with patients exhibiting intensifying symptoms and reduced daily function. These findings suggest a lack of long-term benefit and a potential worsening of the disease burden [59]. Andrashko *et al*. [60] demonstrated that concomitant benzodiazepine use significantly attenuates the antidepressant efficacy of ketamine. Compared to non-users, the co-medication group exhibited lower response rates and a prolonged time to remission. Consequently, benzodiazepines are identified as a negative predictor for ketamine treatment outcomes. Su *et al*. [61] further corroborated the detrimental impact of benzodiazepines on antidepressant efficacy. Notably, even a single dose of ultra-short-acting midazolam can trigger retrograde amnesia-related withdrawal symptoms 4-8 months after use. The discontinuation of benzodiazepines also entails significant risks, such as relapse, rebound phenomena, and symptom worsening [62]. In summary, current evidence indicates that the adverse effects of benzodiazepines in antidepressant therapy significantly outweigh their potential benefits.

## 3 INHALED ANESTHETICS

### 3.1 Sevoflurane

 The mechanism of action of sevoflurane, a standard inhalational anesthetic, remains incompletely understood. S. Wang *et al*. [63] documented a case study involving a patient with MDD lasting 29 years, who had received inadequate treatment with conventional antidepressants and ECT for one month. The patient was transitioned to continuous inhalation of sevoflurane at low concentrations (0.8 to 1.0 mac) *via* facemask for one hour per session, resulting in significant alleviation of depressive symptoms at both two hours and 24 hours post-treatment. Systematic reviews and meta-analyses indicate that administering sevoflurane during ECT reduces seizure duration [64]. Wu *et al*. [65] demonstrated that the antidepressant and anxiolytic effects of inhaled 2.5% sevoflurane administered for 30 minutes were comparable to those of ketamine and persisted for up to 48 hours. Subsequent research indicated that sevoflurane may achieve its rapid and enduring antidepressant effects by modulating neuronal activity in the nucleus accumbens core. A recent preclinical investigation utilizing CUMS modeling in C57BL/6J mice showed that inhalation of 2.5% sevoflurane produced strong antidepressant-like effects. Sevoflurane demonstrated antidepressant-like effects *via* the BDNF-TrkB signaling pathway [11], potentially involving the NMDA receptor [66]. The examination of associated mechanisms remains limited, potentially due to the unclear mechanism of sevoflurane general anesthesia, necessitating much further research to establish a conclusive determination.

### 3.2 Nitrous Oxide (N2O)

N_2_O is a gaseous inhalational anesthetic and NMDAR antagonist commonly used in dental and surgical settings. To produce sedation and pain relief, the drug acts as a broad inhibitor. It blocks central NMDARs and GABARs, while also suppressing spinal opioid receptors and proteins in the descending pathway. Notably, N_2_O has also demonstrated rapid and sustained efficacy in treating patients with depression [67-69]. In a phase II clinical trial [67], 20 patients with TRD were randomly assigned to receive a single 1-hour inhalation of either 50% N_2_O, 25% N_2_O, or placebo (air). The trial results indicated that N_2_O produced a rapid and substantial antidepressant effect in patients with TRD when compared to placebo, with no significant difference in therapeutic efficacy between 25% and 50% N_2_O. However, the side effects associated with inhalation of 25% N_2_O were only one-fourth those of 50% N_2_O, suggesting that 25% N_2_O is the preferred option. In the same research, individuals administered one hour of inhaled 25% N_2_O experienced effects that persisted for a minimum of 24 hours, with depressed symptoms ameliorating over the subsequent two weeks. A double-masked, placebo-controlled trial randomly allocated 23 participants to either the placebo group (11) or the N_2_O group (12). N_2_O was administered at a 50% concentration diluted in 50% oxygen for 60 minutes across eight sessions (twice weekly for four weeks), while the placebo group inhaled 100% oxygen during the same timeframe, with alternating trial intervals of three or four days. The study's findings indicated a more pronounced reduction in the HAM-D17 and N_2_O fractions compared to the placebo group [69].To elucidate the mechanism underlying N_2_O-induced antidepressant effects, W. Liu *et al*. [70] posited that these effects may be mediated by the activation of the neuronal nitric oxide synthase (nNOS)/NO pathway in the mPFC. They tested this hypothesis by administering the NOS inhibitor, L-NG-nitroarginine methyl ester (L-NAME, 10 μg/μl), 30 minutes prior to each N_2_O exposure in the mPFC (0.1 μl/min for 5 minutes), subsequently exposing the mice to a 50% N_2_O and 50% O_2_ mixture for two hours on three consecutive days. The outcomes of subsequent behavioral assessments indicated that repeated N_2_O exposure diminished the immobility duration in the FST, while pretreatment with L-NAME (5 μg/side) administered in the mPFC was observed to reverse the immobility duration in the Tail Suspension Test (TST). This corroborated their hypothesis that the activation of nNOS in the mPFC could elicit an antidepressant-like effect in mice subjected to repeated N_2_O exposure. N_2_O is anticipated to represent a novel strategy for the treatment of depression.

### 3.3 Isoflurane

 Since 1985, numerous papers have suggested the antidepressant benefits of isoflurane [71-73]. A recent preclinical study using a rat LH model demonstrated antidepressant effects of isoflurane following 30 minutes of exposure over six consecutive days, with subsequent investigations revealing the pivotal role of the BDNF-TrkB signaling pathway in this mechanism [74]. A comparable phenomenon was observed in a 2019 study, in which isoflurane effectively mitigated the aberrant BDNF expression elicited by CUMS surgery, activated the TrkB signaling pathway, and facilitated the development of dendritic spines in the mPFC, CA1, CA3, and DG [73]. Overall, the existing literature regarding the effects of isoflurane on depression remains limited and requires further empirical substantiation.

## 4 ANALGESICS

### 4.1 Opioids

The endogenous opioid system comprises various opioid neuropeptides and receptors, with the three primary opioid receptors-μ-opioid receptors (MOR), κ-opioid receptors (KOR), and δ-opioid receptors (DOR)-serving as the principal targets of opioid action. These receptor systems are directly implicated in mood regulation and exhibit dysregulation in depression [75-77].

#### 4.1.1 MOR

In 1996, it was reported that the MOR agonist morphine exhibited effects akin to TCAs in mitigating depression-related behaviors in rats [78], a finding corroborated by subsequent animal studies indicating that morphine decreased immobility time in the TST [79] and FST [80]. Furthermore, MOR knockout mice demonstrated reduced motivation to eat [81] and diminished social interactions [75] with these behavioral alterations closely linked to depression, implying that MOR plays a beneficial role in preventing the onset of depression. Karp *et al*. [82] conducted an 8-week clinical trial of buprenorphine (KOR antagonist, MOR agonist; mean dose, 0.4 mg/day) involving 13 patients with TRD aged 50 years or older. They defined a Montgomery-Åsberg Depression Rating Scale (MADRS) score of ≤10 as symptomatic remission. The results indicated that 8 participants met the remission criterion at 8 weeks, demonstrating the substantial antidepressant effect of MOR. Early mechanistic studies revealed that MOR activation in the DRN disinhibits serotonergic neurons by suppressing GABAergic inputs. This process leads to an excessive release of serotonin in the PFC and other mood-regulating regions, such as the thalamus, HC, NAc, amygdala, and striatum [75, 83, 84]. This pertains to the therapeutic mechanisms of depression, indicating that this may represent a potential mechanism of MOR antidepressants. Furthermore, a recent, extensive, retrospective cross-sectional study demonstrated that subclinical depression and anxiety symptoms correlated with diminished MOR availability in cortical and subcortical regions, including the amygdala, insula, HC, ventral striatum, orbitofrontal cortex, and cingulate cortex [77]. Preclinical mechanistic investigations have further established MOR as a novel therapeutic approach for MDD.

#### 4.1.2 KOR

 KOR agonists, on the other hand, have been shown to have pro-depressant effects [85]. Reports indicate that KOR activation reduces dopamine release within mesolimbic structures, thereby directly inhibiting the brain’s reward pathway. This suppression creates a negative emotional state resembling depression [86, 87]. However, some recent clinics have suggested the opposite. Fifty mice were exposed to CUMS for 4 weeks and given the selective KOR antagonist Aticaprant (10 mg/kg, i.p.) daily starting in the last week of CUMS, followed by behavioral tests such as SPT, nest construction, FST, and hot plate test. The results showed that single or multiple treatments of Aticaprant reversed most of the behavioral test effects induced by CUMS [88]. A phase 2, randomized, double-blind study evaluated Aticaprant (10 mg) as an adjunctive therapy in MDD patients with inadequate response to SSRIs/SNRIs. The results showed that adjunctive Aticaprant significantly reduced depressive symptoms compared to placebo [89]. Despite unclear mechanisms, a single dose of nor-BNI (20 μg) was found to reduce immobility time on day 1, concurrent with a surge in BDNF mRNA in the HC, amygdala, and PFC. Moreover, the persistence of these antidepressant-like effects was synchronized with BDNF mRNA upregulation. This suggests that BDNF signaling is pivotal to the antidepressant properties of KOR antagonists [90]. Collectively, current research establishes that KOR activation exerts pro-depressive effects, whereas KOR antagonism elicits robust antidepressant efficacy.

#### 4.1.3 DOR

 In animal models, the DOR agonist SNC80 reversed depressive-like behaviors in CUMS mice by specifically upregulating hippocampal BDNF/TrkB signaling. These findings indicate that DOR agonists possess potential antidepressant activity [91]. A novel DOR agonist, KNT-127, demonstrated antidepressant-like and anti-injurious effects in mice. A comparison of subcutaneous injections of KNT-127 and SNC80 indicated that mice experienced transient convulsive episodes lasting 10-15 seconds approximately 10 minutes post-injection of SNC80 (30 mg/kg), whereas high-dose (100 mg/kg) injections of KNT-127 did not induce convulsions or rigidity [92]. Yoshioka *et al*. recently established that KNT-127 exhibits fewer adverse effects as a DOR agonist in the treatment of depression [93]. Overall, DOR has a contributory role in antidepressants. DOR agonists have been shown to increase BDNF mRNA expression in various brain areas in rats [76]. Torregrossa *et al*. discovered that a high-dose DOR agonist (DPDPE, 155 nmol) elicited strong antidepressant-like effects in the FST and noted that this dosage significantly elevated BDNF mRNA expression in the frontal cortex and the hippocampal CA1 region of rats [94]. These findings further substantiate the therapeutic potential of DORs in depression.

Despite increasing data regarding the possible therapeutic advantages of opioids for individuals with depression, the Miami University study showed that opioids can enhance short-term mood. Nonetheless, prolonged usage (exceeding 30 days) presents a danger of developing new-onset depression [95]. The researchers determined that prolonged opioid usage may modify the structure of the nervous system, with the risk escalating from 1.18 to 1.33 for usage between 31 and 90 days, and further increasing for usage exceeding 90 days [96]. Another study identified MDD as a potential causal risk factor for increased prescription opioid use. This implies that patients with depression are more susceptible to opioid misuse or dependence, and that opioid use may, in turn, exacerbate depressive symptoms [97]. The impact of opioids on depression is likely bidirectional. Certain opioids may positively influence depression treatment under specific conditions; conversely, prolonged opioid usage may elevate the risk of depression. Consequently, while administering opioids, it is imperative to evaluate the risks and benefits for the patient meticulously and to formulate a personalized treatment strategy.

### 4.2 Tramadol

 Tramadol is a centrally acting analgesic primarily used for the management of moderate to severe pain. Its mechanism of action involves both opioid and non-opioid pathways. Tramadol exhibits structural and functional parallels with the antidepressant venlafaxine, and a case report detailing severe depressive symptoms following the cessation of tramadol in a patient with prolonged use indicates that tramadol may contribute to the prevention of depression [98]. Research indicates that tramadol PCIA diminishes the prevalence of depression during the early postpartum phase [99]. Additionally, administering tramadol at a dosage of 40 mg/kg ameliorated depression-like behavior in mice within a behavioral despair model [100]. Tramadol operates through a dual mechanism: it elevates monoamine neurotransmitter levels in the synaptic cleft by inhibiting the reuptake of norepinephrine and serotonin in neurons, thereby increasing synaptic concentrations of 5-HT and NE, which is fundamental to depression treatment. Arakawa *et al*. [101] conducted Positron Emission Tomography (PET) using [11C] MADAM and [18F] FMeNER-D2 in six cynomolgus monkeys following intravenous tramadol administration (1, 2, and 4 mg/kg). Results showed that at the 4 mg/kg dose, occupancy of both 5-HTT and NET exceeded 70%. This confirms that tramadol effectively inhibits reuptake and boosts synaptic 5-HT and NE availability, thereby exerting an antidepressant effect. Furthermore, its antidepressant mechanism may involve the suppression of NMDA receptors [100]. Additionally, tramadol interacts with opioid receptors to elicit analgesia, drowsiness, and pleasure. In a chronic constriction injury model of neuropathic pain in rats, tramadol was observed to ameliorate depressive behavior following nerve injury, potentially attributable to its analgesic and antidepressant properties, thereby mitigating depressive symptoms [102]. However, prolonged high-dose reliance on tramadol may precipitate depression [103], possibly linked to the reduction in endogenous opioid release from the central nervous system due to long-term tramadol use, culminating in heightened tolerance and dependence. Such dependence is associated with increased tolerance, dysphoria, lethargy, anxiety, and insomnia. Since these symptoms mimic the clinical presentation of depression, they may potentially exacerbate the patient’s condition [104].

## CONCLUSION

 While the precise roots of both general anesthesia and depression remain somewhat elusive, their biological pathways clearly converge on similar neural networks. This overlap provides a compelling rationale for the growing focus on anesthetics as a novel frontier in antidepressant therapy Fig. (**1**). Ketamine significantly alleviates serious depression and plays a crucial role in advancing anesthetic therapies. Current research has validated the beneficial impact of certain general anesthetics on alleviating depressed symptoms, including dexmedetomidine, various opioids, and N_2_O. The evidence presented by pertinent studies is steadily accumulating (Table **1**). Simultaneously, prolonged administration of anesthetic agents may induce or exacerbate pre-existing depressed symptoms, therefore necessitating an urgent elucidation of the mechanisms by which antidepressants interact with general anesthetics. In summary, the interaction between general anesthetics and depression constitutes a complex and multifaceted research landscape. Translating these mechanistic insights into clinical practice represents our best chance to overcome the stagnation in antidepressant therapy. By targeting rapid-acting pathways, we can finally offer patients relief without the debilitating side effects and weeks of delay inherent to standard care. Therefore, exploring novel applications of general anesthetics not only offers fresh strategies for treating depression but also identifies new targets for future drug development.

## Figures and Tables

**Fig. (1):**
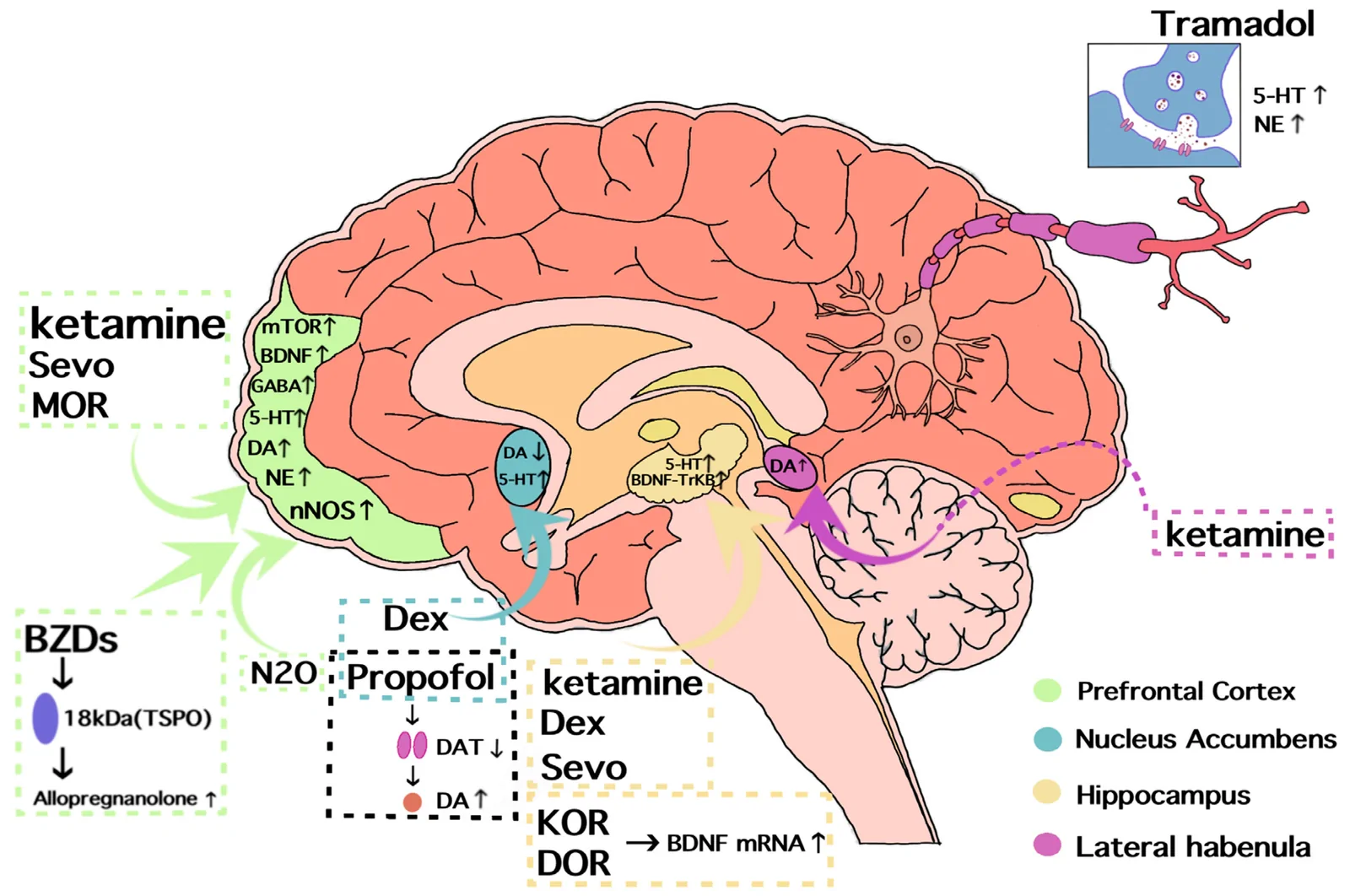
Prefrontal Cortex (PFC, Green Zone): Multiple agents, including Ketamine, Sevoflurane (Sevo), Nitrous Oxide (N2O), and μ-opioid receptor (MOR) agonists, converge in the PFC to enhance synaptic plasticity and neurotransmission. This is characterized by the upregulation of the mTOR signaling pathway, BDNF expression, nNOS activity, and extracellular levels of monoamines. Nucleus Accumbens (NAc, Blue Zone): Propofol and Dex modulate the reward circuitry within the NAc. Propofol inhibits the Dopamine Transporter (DAT), leading to altered synaptic DA and 5-HT dynamics. Hippocampus (HC, Yellow Zone): Ketamine, Dexmedetomidine (Dex), Sevo, and κ-/δ-opioid receptor (KOR/DOR) agonists converge to promote neurotrophic signaling. These agents increase BDNF mRNA expression, thereby enhancing downstream BDNF-TrkB signaling cascades and elevating 5-HT levels, which are critical for hippocampal neurogenesis and synaptic remodeling. Lateral Habenula (LHb, Purple Zone) & Other Mechanisms: Ketamine uniquely modulates neuronal activity in the LHb to influence downstream dopaminergic output. The inset (top right) illustrates Tramadol, which functions *via* dual inhibition of 5-HT and NE reuptake. Dexmedetomidine (Dex); Benzodiazepines (BZDs); Sevoflurane (Sevo); μ-opioid receptor (MOR); κ-opioid receptor (KOR); Delta-opioid receptor (DOR).

**Table 1:** The effects of general anesthetic agents on depression and their possible mechanisms.

**Category**	**Drug Name**	**Effect on Depression: Potential Mechanisms and Pathways**		**Clinical Research**
**Intravenous Anesthetics**	1.1 Ketamine	Significant rapid antidepressant	1) **Block NMDA receptors:** inhibition of GABAergic interneurons leads to a disinhibition-mediated glutamate burst, which induces the formation of dendritic spines and exerts rapid antidepressant effects [23]. 2) Suppression of burst firing in the **LHb** relieves inhibition of downstream dopaminergic and serotonergic neurons, thereby rapidly ameliorating depressive symptoms [26]. 3) **AMPA receptor activation:** upregulates BDNF expression and activates the BDNF-TrkB pathway, promoting hippocampal neurogenesis and enhancing fEPSP [27-29]. 4) **Modulation of inflammatory and mTORC1 signaling:** Downregulating proinflammatory cytokines while upregulating the mTORC1 pathway drives synaptic protein synthesis, thereby enhancing synaptic plasticity [31, 32].	**Clinical: “Gold Standard”:** Sub-anesthetic dose (0.5mg/kg) improves TRD and suicidal ideation within hours; lasts days to weeks [16, 20]. S-ketamine is FDA-approved.
	1.2 Dexmedetomidine	Adjunctive antidepressant	1) **α2-Adrenergic receptor agonism:** Inhibits sympathetic outflow and attenuates the physiological stress response [44]. 2) **Upregulation of DA receptors:** Stimulates 5-HT synthesis in the DRN and reduces dopamine levels in the NAc, leading to a compensatory upregulation of dopamine receptors in mood-regulating regions [49]. 3) **Promotion of neurogenesis:** Alleviates depressive phenotypes by enhancing neurogenesis specifically in the hippocampal dentate gyrus [50].	**Clinical Adjunctive:** Erioperative use reduces POPD and postpartum depression (meta-analyses) [47, 48]. Few studies have been conducted as monotherapy for MDD。
	1.3 Propofol	Mixed effects	1) **Targeting dopamine transporters (DAT):** Increases dopamine levels in the NAc to activate reward pathways, thereby inducing euphoria and ameliorating anhedonia [53]. 2) **CaMKIIα pathway activation:** Upregulates hippocampal PSD-95 and GluA1 expression to restore synaptic plasticity, effectively reversing depressive behaviors induced by learned helplessness [54].	**Clinical Controversial results:** Deep anesthesia may mimic ECT effects [51]. Some studies show no effect or worsening [56]. Used for ECT induction.
	1.4 Benzodiazepines	Mixed effects	1) **Allosteric modulation of GABA-A receptors:** Inhibits neuronal excitability through receptor modulation [57]. 2) **Neurosteroid biosynthesis:** Potentially mediated *via* TSPO-stimulated biosynthesis of allopregnanolone in the PHC and HC [58].	**Clinical Adjunctive:** May attenuate ketamine’s efficacy. Long-term use linked to increased depression risk [59, 61].
**Inhalation Anesthetics**	2.1 Sevoflurane	Potential rapid antidepressant.	1) **Activation of GABAergic neurons:** Stimulates GABAergic neurons in the NAc, thereby suppressing excessive glutamate release [65]. 2) **BDNF pathway enhancement:** Increases BDNF expression, promoting neuroplasticity and functional recovery [66].	**Clinical Exploratory phase:** Inhalation may benefit depression; compared to propofol-ECT, may shorten recovery time and improve cognition [64].
	2.2 Nitrous Oxide (N_2_O)	Rapid antidepressant	1) **Increasing mPFC excitability:** Enhances BDNF expression levels and burst firing rates in an nNOS-activation-dependent manner [70].	**Clinical Growing evidence:**50% N_2_O in TRD shows a rapid effect; fewer side effects than ketamine, but variable duration [68].
	2.3 Isoflurane	Mixed effects	1) **TrkB signaling activation:** Induces neurogenesis by promoting dendritic spine formation in the mPFC, CA1, CA3, and DG regions [73].	**Clinical Preliminary:** Effective in animal models. Small samples suggest potential to replace ECT; tolerable, with symptom improvement [72].
**Analgesics**	3.1 Opioids μ-opioid receptors (MOR)	Short-term euphoria Long-term depression	1) **MOR activation:** Stimulates receptors in the DRN to suppress GABA-mediated inhibition of serotonergic neurons, thereby modulating excessive serotonin 5-HT release in the PFC and other mood-regulating regions [83, 84].	**Clinical Early phase**： The MOR agonist buprenorphine (0.4 mg/day) achieved remission criteria within 8 weeks [75].
	κ-opioid receptors(KOR)	Agonists induce depression. Antagonists exhibit antidepressant effects.	1) **KOR activation:** Inhibits dopamine release, thereby mediating stress-induced depressive states and anhedonia [87]. 2) **KOR antagonism:** Restores reward circuitry function and upregulates BDNF mRNA expression, demonstrating antidepressant potential [90].	**Clinical R&D Hotspot:** Antagonists (*e.g*., Aticaprant) in trials for anhedonia [89].
	δ-opioid receptors (DOR)	Potential antidepressant efficacy	1) **DOR activation:** Modulates the hippocampal BDNF/TrkB signaling pathway to promote neurogenesis [91].	**Primarily Preclinical:** DOR agonists demonstrate antidepressant activity and elevate BDNF levels in animal models; however, large-scale clinical translation is currently lacking [76].
	3.2 Tramado	Mixed effects	1) **SNRI mechanism:** Inhibits the synaptic reuptake of serotonin 5-HT and NE, resulting in elevated concentrations within the synaptic cleft [100].	**Clinical Controversial:** Rapid effect in animals. Case reports for TRD, but addiction/seizure risks limit routine recommendation [104].

## LIST OF ABBREVIATIONS

AMPA: α-Amino-3-hydroxy-5-Methyl-4-isoxazolepropionic Acid
AMSP: European Drug Monitoring Program
BDI: Beck Depression Inventory
BDNF: Brain-derived Neurotrophic Factor
BZDs: Benzodiazepines
cLH: Congenitally Learned Helpless
CUMS: Chronic Unpredictable Mild Stress
DEX: Dexmedetomidine
DOR: δ-Opioid Receptor
ECT: Electroconvulsive Treatment
fEPSPs: Field Excitatory Postsynaptic Potentials
FST: Forced Swimming Test
HC: Hippocampus
HDRS: Hamilton Depression Rating Scale
HDRS-24: 24-item Hamilton Depression Rating Scale
KOR: Κ-Opioid Receptor
LH: Learned Helplessness
LHb: Lateral Habenula
L-NAME: L-NG-nitroarginine Methyl Ester
MDD: Major Depressive Disorder
MOR: μ-Opioid Receptor
mPFC: Medial Prefrontal Cortex
NHANES: National Health and Nutrition Examination Survey
NIMH: National Institute of Mental Health
NMDA: N-Methyl-D-Aspartate
nNOS: Neuronal Nitric Oxide Synthase
PET: Positron Emission Tomography
PFC: Prefrontal Cortex
PPD: Postpartum Depression
SPT: Sucrose Preference Test
SSRIs: Selective Serotonin Reuptake Inhibitors
TCAs: Tricyclic Antidepressants
TRD: Treatment-Refractory Depression
TST: Tail Suspension Test
α2-AR: α2-Adrenergic Receptor

## References

[1] Thati R.P., Dhadwal A.S., Kumar P., P S. (2023). A novel multi-modal depression detection approach based on mobile crowd sensing and task-based mechanisms.. Multimedia Tools Appl..

[2] McIntyre R.S., Alsuwaidan M., Baune B.T., Berk M., Demyttenaere K., Goldberg J.F., Gorwood P., Ho R., Kasper S., Kennedy S.H., Ly-Uson J., Mansur R.B., McAllister-Williams R.H., Murrough J.W., Nemeroff C.B., Nierenberg A.A., Rosenblat J.D., Sanacora G., Schatzberg A.F., Shelton R., Stahl S.M., Trivedi M.H., Vieta E., Vinberg M., Williams N., Young A.H., Maj M. (2023). Treatment-resistant depression: Definition, prevalence, detection, management, and investigational interventions.. World Psychiatry.

[3] Trivedi M.H., Rush A.J., Wisniewski S.R., Nierenberg A.A., Warden D., Ritz L., Norquist G., Howland R.H., Lebowitz B., McGrath P.J., Shores-Wilson K., Biggs M.M., Balasubramani G.K., Fava M. (2006). Evaluation of outcomes with citalopram for depression using measurement-based care in STAR*D: Implications for clinical practice.. Am J. Psychiatry.

[4] Blackburn T.P. (2019). Depressive disorders: Treatment failures and poor prognosis over the last 50 years.. Pharmacol. Res. Perspect..

[5] Solmi M., Fornaro M., Ostinelli E.G., Zangani C., Croatto G., Monaco F., Krinitski D., Fusar-Poli P., Correll C.U. (2020). Safety of 80 antidepressants, antipsychotics, anti-attention-deficit/hyperactivity medications and mood stabilizers in children and adolescents with psychiatric disorders: A large scale systematic meta-review of 78 adverse effects.. World Psychiatry.

[6] Meshkat S., Duffy F.S., Tassone K.V., Lin Q., Ym P.H., Jung H., Lou W., Bhat V. (2025). Increased odds of metabolic syndrome among adults with depressive symptoms or antidepressant use.. Transl. Psychiatry.

[7] El Asmar K., Annan N.B., Khoury R., Colle R., Martin S., Ghoul T.E., Trabado S., Chanson P., Feve B., Verstuyft C., Becquemont L., Corruble E. (2023). Non-overweight depressed patients who respond to antidepressant treatment have a higher risk of later metabolic syndrome: Findings from the METADAP cohort.. Psychol. Med..

[8] Stübner S., Grohmann R., Greil W., Zhang X., Müller-Oerlinghausen B., Bleich S., Rüther E., Möller H.J., Engel R., Falkai P., Toto S., Kasper S., Neyazi A. (2018). Suicidal ideation and suicidal behavior as rare adverse events of antidepressant medication: Current report from the AMSP multicenter drug safety surveillance project.. Int. J. Neuropsychopharmacol..

[9] Berman R.M., Cappiello A., Anand A., Oren D.A., Heninger G.R., Charney D.S., Krystal J.H. (2000). Antidepressant effects of ketamine in depressed patients.. Biol. Psychiatry.

[10] Modir H., Mahmoodiyeh B., Shayganfard M., Abdus A., Almasi-Hashiani A. (2023). Efficacy of ketamine, propofol, and dexmedetomidine for anesthesia in electroconvulsive therapy in treatment-resistant major depressive disorder patients.. Med. Gas Res..

[11] You S., Wu Y., Guo Y., Wu M., Ran M., Cao F., Hao X., Yang L., Zhang H., Mi W., Tong L. (2024). Sevoflurane exerts antidepressant-like effects *via* the BDNF-TrkB pathway.. Behav. Brain Res..

[12] Barra M.E., Solt K., Yu X., Edlow B.L. (2024). Restoring consciousness with pharmacologic therapy: Mechanisms, targets, and future directions.. Neurotherapeutics.

[13] Zarate C.A., Singh J.B., Carlson P.J., Brutsche N.E., Ameli R., Luckenbaugh D.A., Charney D.S., Manji H.K. (2006). A randomized trial of an N-methyl-D-aspartate antagonist in treatment-resistant major depression.. Arch. Gen. Psychiatry.

[14] Lan X., Wang C., Zhang F., Liu H., Li W., Ye Y., Hu Z., Mai S., Ning Y., Zhou Y. (2023). Short-term cognitive effects of repeated-dose esketamine in adolescents with major depressive disorder and suicidal ideation: A randomized controlled trial.. Child Adolesc. Psychiatry Ment. Health..

[15] Wang C., Lan X., Liu W., Zhan Y., Zheng W., Chen X., Liu G., Mai S., Lu H., McIntyre R.S., Zhou Y., Ning Y. (2024). Non-improvement predicts subsequent non-response to repeated-dose intravenous ketamine for depression: A re-analysis of a 2-week open-label study in patients with unipolar and bipolar depression.. Transl. Psychiatry.

[16] Yan R., Marshall T., Khullar A., Nagle T., Knowles J., Malkin M., Chubbs B., Swainson J. (2024). Patient-reported outcomes on sleep quality and circadian rhythm during treatment with intravenous ketamine for treatment-resistant depression.. Ther. Adv. Psychopharmacol..

[17] Chaudhary P., Shah P., Mehta P. (2023). Retrospective data analysis to determine the effectiveness of intravenous ketamine therapy on patients suffering from depression with suicidal ideation.. Ind. Psychiatry J..

[18] Phillips J.L., Norris S., Talbot J., Birmingham M., Hatchard T., Ortiz A., Owoeye O., Batten L.A., Blier P. (2020). Single, repeated, and maintenance ketamine infusions for treatment-resistant depression: A randomized controlled trial.. Focus Am. Psychiatr. Publ..

[19] Loo C., Glozier N., Barton D., Baune B.T., Mills N.T., Fitzgerald P., Glue P., Sarma S., Galvez-Ortiz V., Hadzi-Pavlovic D., Alonzo A., Dong V., Martin D., Nikolin S., Mitchell P.B., Berk M., Carter G., Hackett M., Leyden J., Hood S., Somogyi A.A., Lapidus K., Stratton E., Gainsford K., Garg D., Thornton N.L.R., Fourrier C., Richardson K., Rozakis D., Scaria A., Mihalopoulos C., Chatterton M.L., McDonald W.M., Boyce P., Holtzheimer P.E., Kozel F.A., Riva-Posse P., Rodgers A. (2023). Efficacy and safety of a 4-week course of repeated subcutaneous ketamine injections for treatment-resistant depression (KADS study): Randomised double-blind active-controlled trial.. Br. J. Psychiatry.

[20] Glue P., Neehoff S., Beaglehole B., Shadli S., McNaughton N., Hughes-Medlicott N.J. (2024). Ketamine for treatment-resistant major depressive disorder: Double-blind active-controlled crossover study.. J. Psychopharmacol..

[21] Zanos P., Brown K.A., Georgiou P., Yuan P., Zarate C.A., Thompson S.M., Gould T.D. (2023). NMDA receptor activation-dependent antidepressant-relevant behavioral and synaptic actions of ketamine.. J. Neurosci..

[22] Ago Y., Tanabe W., Higuchi M., Tsukada S., Tanaka T., Yamaguchi T., Igarashi H., Yokoyama R., Seiriki K., Kasai A., Nakazawa T., Nakagawa S., Hashimoto K., Hashimoto H. (2019). (R)-ketamine induces a greater increase in prefrontal 5-HT release than (S)-ketamine and ketamine metabolites *via* an AMPA receptor-independent mechanism.. Int. J. Neuropsychopharmacol..

[23] Wu M., Minkowicz S., Dumrongprechachan V., Hamilton P., Kozorovitskiy Y. (2021). Ketamine rapidly enhances glutamate-evoked dendritic spinogenesis in medial prefrontal cortex through dopaminergic mechanisms.. Biol. Psychiatry.

[24] Nicosia N., Giovenzana M., Misztak P., Mingardi J., Musazzi L. (2024). Glutamate-mediated excitotoxicity in the pathogenesis and treatment of neurodevelopmental and adult mental disorders.. Int. J. Mol. Sci..

[25] Yang Y., Cui Y., Sang K., Dong Y., Ni Z., Ma S., Hu H. (2018). Ketamine blocks bursting in the lateral habenula to rapidly relieve depression.. Nature.

[26] Ma S., Chen M., Jiang Y., Xiang X., Wang S., Wu Z., Li S., Cui Y., Wang J., Zhu Y., Zhang Y., Ma H., Duan S., Li H., Yang Y., Lingle C.J., Hu H. (2023). Sustained antidepressant effect of ketamine through NMDAR trapping in the LHb.. Nature.

[27] Freudenberg F., Reif-Leonhard C., Dawson G.R., McKernan R.M., Reif A. (2025). All roads lead to glutamate: NMDA and AMPA receptors as targets for rapid-acting antidepressants.. Pharmacol. Res..

[28] Li S., Luo X., Hua D., Wang Y., Zhan G., Huang N., Jiang R., Yang L., Zhu B., Yuan X., Luo A., Yang C. (2020). Ketamine alleviates postoperative depression-like symptoms in susceptible mice: The role of BDNF-TrkB signaling.. Front. Pharmacol..

[29] Lin P.Y., Ma Z.Z., Mahgoub M., Kavalali E.T., Monteggia L.M. (2021). A synaptic locus for TrkB signaling underlying ketamine rapid antidepressant action.. Cell. Rep..

[30] Tan J.Q., Gu L.M., Zhou Y.L., Wang C.Y., Lan X.F., Zheng W., Ning Y.P. (2025). Body mass index is associated with the antidepressant effects of intravenous ketamine in patients with depression.. Front. Psychiatry.

[31] Nikkheslat N. (2021). Targeting inflammation in depression: Ketamine as an anti-inflammatory antidepressant in psychiatric emergency.. Brain Behav. Immun. Health.

[32] Kopra E., Mondelli V., Pariante C., Nikkheslat N. (2021). Ketamine's effect on inflammation and kynurenine pathway in depression: A systematic review.. J. Psychopharmacol..

[33] Garro-Martínez E., Fullana M.N., Florensa-Zanuy E., Senserrich J., Paz V., Ruiz-Bronchal E., Adell A., Castro E., Díaz Á., Pazos Á., Bortolozzi A., Pilar-Cuéllar F. (2021). mTOR knockdown in the infralimbic cortex evokes a depressive-like state in mouse.. Int. J. Mol. Sci..

[34] Johnston J.N., Thacker J.S., Desjardins C., Kulyk B.D., Romay-Tallon R., Kalynchuk L.E., Caruncho H.J. (2020). Ketamine rescues hippocampal reelin expression and synaptic markers in the repeated-corticosterone chronic stress paradigm.. Front. Pharmacol..

[35] Marcatili M., Sala C., Dakanalis A., Colmegna F., D’Agostino A., Gambini O., Dell’Osso B., Benatti B., Conti L., Clerici M. (2020). Human induced pluripotent stem cells technology in treatment resistant depression: Novel strategies and opportunities to unravel ketamine’s fast-acting antidepressant mechanisms.. Ther. Adv. Psychopharmacol..

[36] Abdallah C.G., Averill L.A., Gueorguieva R., Goktas S., Purohit P., Ranganathan M., Sherif M., Ahn K.H., D'Souza D.C., Formica R., Southwick S.M., Duman R.S., Sanacora G., Krystal J.H. (2020). Modulation of the antidepressant effects of ketamine by the mTORC1 inhibitor rapamycin.. Neuropsychopharmacology.

[37] Ji N., Lei M., Chen Y., Tian S., Li C., Zhang B. (2023). How oxidative stress induces depression?. ASN Neuro.

[38] Zhou N., Shi X., Wang R., Wang C., Lan X., Liu G., Li W., Zhou Y., Ning Y. (2025). Proteomic patterns associated with ketamine response in major depressive disorders.. Cell. Biol. Toxicol..

[39] De Berardis D., Fornaro M., Valchera A., Cavuto M., Perna G., Di Nicola M., Serafini G., Carano A., Pompili M., Vellante F., Orsolini L., Fiengo A., Ventriglio A., Yong-Ku K., Martinotti G., Di Giannantonio M., Tomasetti C. (2018). Eradicating suicide at its roots: Preclinical bases and clinical evidence of the efficacy of ketamine in the treatment of suicidal behaviors.. Int. J. Mol. Sci..

[40] Carlessi A.S., Botelho M.E.M., Manosso L.M., Borba L.A., Maciel L.R., Andrade N.M., Martinello N.S., Padilha A.P.Z., Generoso C.M., Bencke C.V., de Moura A.B., Lodetti B.F., Collodel A., Joaquim L., Bonfante S., Biehl E., Generoso J.S., Arent C.O., Barichello T., Petronilho F., Quevedo J., Réus G.Z. (2022). Sex differences on the response to antidepressants and psychobiotics following early life stress in rats.. Pharmacol. Biochem. Behav..

[41] Abelaira H.M., Veron D.C., de Moura A.B., Carlessi A.S., Borba L.A., Botelho M.E.M., Andrade N.M., Martinello N.S., Zabot G.C., Joaquim L., Biehl E., Bonfante S., Budni J., Petronilho F., Quevedo J., Réus G.Z. (2021). Sex differences on the behavior and oxidative stress after ketamine treatment in adult rats subjected to early life stress.. Brain Res. Bull.

[42] Muscat S.A., Hartelius G., Crouch C.R., Morin K.W. (2021). An integrative approach to ketamine therapy may enhance multiple dimensions of efficacy: Improving therapeutic outcomes with treatment resistant depression.. Front. Psychiatry.

[43] Chubbs B., Wang J., Archer S., Chrenek C., Khullar A., Wolowyk M., Swainson J. (2022). A survey of drug liking and cravings in patients using sublingual or intranasal ketamine for treatment resistant depression: A preliminary evaluation of real world addictive potential.. Front. Psychiatry.

[44] Guan Z.H., Yang D., Wang Y., Ma J.B., Wang G.N. (2024). Ectodysplasin-A2 receptor (EDA2R) knockdown alleviates myocardial ischemia/reperfusion injury through inhibiting the activation of the NF-κB signaling pathway.. Exp. Anim..

[45] Shams T., El-Masry R. (2014). Ketofol-Dexmedetomidine combination in ECT: A punch for depression and agitation.. Indian J. Anaesth..

[46] Liu Y., Hu Q., Xu S., Li W., Liu J., Han L., Mao H., Cai F., Liu Q., Zhu R., Fang C., Lou Y., Wang Z., Yang H., Wang W. (2024). Antidepressant effects of dexmedetomidine compared with ECT in patients with treatment-resistant depression.. J. Affect Disord..

[47] Xu S., Zhou Y., Wang S., Li Q., Feng Y., Chen L., Duan K. (2024). Perioperative intravenous infusion of dexmedetomidine for alleviating postpartum depression after cesarean section: A meta-analysis and systematic review.. Eur. J. Obstet. Gynecol. Reprod. Biol..

[48] Zhou Y., Bai Z., Zhang W., Xu S., Feng Y., Li Q., Li L., Ping A., Chen L., Wang S., Duan K. (2024). Effect of dexmedetomidine on postpartum depression in women with prenatal depression: A randomized clinical trial.. JAMA Netw Open.

[49] Moon E.J., Ko I.G., Kim S.E., Jin J.J., Hwang L., Kim C.J., An H., Lee B.J., Yi J.W. (2018). Dexmedetomidine ameliorates sleep deprivation-induced depressive behaviors in mice.. Int. Neurourol. J..

[50] Xu S., Zhao X., Zhu Z., He M., Zheng X., Zhang X. (2022). A new potential antidepressant: Dexmedetomidine alleviates neuropathic pain-induced depression by increasing neurogenesis in the hippocampus.. Pharmacology.

[51] Zeng Q.B., Zou D.C., Huang X.B., Shang D.W., Huang X., Yang X.H., Ning Y.P., Balbuena L., Xiang Y.T., Zheng W. (2025). Efficacy and safety of esketamine versus propofol in electroconvulsive therapy for treatment-resistant depression: A randomized, double-blind, controlled, non-inferiority trial.. J. Affect Disord..

[52] Mickey B.J., White A.T., Arp A.M., Leonardi K., Torres M.M., Larson A.L., Odell D.H., Whittingham S.A., Beck M.M., Jessop J.E., Sakata D.J., Bushnell L.A., Pierson M.D., Solzbacher D., Kendrick E.J., Weeks H.R., Light A.R., Light K.C., Tadler S.C. (2018). Propofol for treatment-resistant depression: A pilot study.. Int. J. Neuropsychopharmacol..

[53] Zhu X.N., Li J., Qiu G.L., Wang L., Lu C., Guo Y.G., Yang K.X., Cai F., Xu T., Yuan T.F., Hu J. (2023). Propofol exerts anti-anhedonia effects *via* inhibiting the dopamine transporter.. Neuron.

[54] Wang J., Xiang J., Han X., Liang J., Shang X. (2025). Propofol alleviates depression-like behavior and cognitive disorder in learned helplessness model mice *via* regulating synaptic function.. J. Neurosci. Res..

[55] Song F., Lv X., Meng J. (2019). Propofol induces postoperative depression and inhibits microglial function in mice.. Mediators Inflamm..

[56] Daniel D.G., Daniel N.G., Daniel D.T., Flynn L.C., Allen M.H. (2020). The effect of propofol on a forced swim test in mice at 24 hours.. Curr. Ther. Res. Clin. Exp..

[57] Shiwaku H., Fujita M., Takahashi H. (2020). Benzodiazepines reduce relapse and recurrence rates in patients with psychotic depression.. J. Clin. Med..

[58] Qiu Z.K., Li M.S., He J.L., Liu X., Zhang G.H., Lai S., Ma J.C., Zeng J., Li Y., Wu H.W., Chen Y., Shen Y.G., Chen J.S. (2015). Translocator protein mediates the anxiolytic and antidepressant effects of midazolam.. Pharmacol. Biochem. Behav..

[59] Fond G., Faugere M., Boyer L., Peri P., Stephan F., Moliere F., Anguill L., Bennabi D., Haffen E., Bouvard A., Walter M., Samalin L., Llorca P.M., Genty J.B., Leboyer M., Holtzmann J., Nguon A.S., Rey R., Horn M., Vaiva G., Hennion V., Etain B., El-Hage W., Camus V., Courtet P., Aouizerate B., Yrondi A., Lancon C., Richieri R. (2023). Long-term benzodiazepine prescription in treatment-resistant depression: A national FACE-TRD prospective study.. Prog. Neuropsychopharmacol. Biol. Psychiatry.

[60] Andrashko V., Novak T., Brunovsky M., Klirova M., Sos P., Horacek J. (2020). The antidepressant effect of ketamine is dampened by concomitant benzodiazepine medication.. Front. Psychiatry.

[61] Su T.P., Li C.T., Lin W.C., Wu H.J., Tsai S.J., Bai Y.M., Mao W.C., Tu P.C., Chen L.F., Li W.C., Chen M.H. (2023). A randomized, double-blind, midazolam-controlled trial of low-dose ketamine infusion in patients with treatment-resistant depression and prominent suicidal ideation.. Int. J. Neuropsychopharmacol..

[62] Cosci F., Chouinard G. (2020). Acute and persistent withdrawal syndromes following discontinuation of psychotropic medications.. Psychother. Psychosom..

[63] Wang S., Cheng S., Feng M., Guo P., Qian M., Shen X., Chen R., Wang G. (2020). Sevoflurane augmentation in treatment-resistant depression: A clinical case study.. Ther. Adv. Psychopharmacol..

[64] Aoki N., Suwa T., Kawashima H., Tajika A., Sunada N., Shimizu T., Murai T., Kinoshita T., Takekita Y. (2021). Sevoflurane in electroconvulsive therapy: A systematic review and meta-analysis of randomised trials.. J. Psychiatr. Res..

[65] Wu M., Li A., Guo Y., Cao F., You S., Cao J., Mi W., Tong L. (2023). GABAergic neurons in the nucleus accumbens core mediate the antidepressant effects of sevoflurane.. Eur. J. Pharmacol..

[66] Guo F., Zhang B., Shen F., Li Q., Song Y., Li T., Zhang Y., Du W., Li Y., Liu W., Cao H., Zhou X., Zheng Y., Zhu S., Li Y., Liu Z. (2024). Sevoflurane acts as an antidepressant by suppression of GluN2D-containing NMDA receptors on interneurons.. Br. J. Pharmacol..

[67] Nagele P., Palanca B.J., Gott B., Brown F., Barnes L., Nguyen T., Xiong W., Salloum N.C., Espejo G.D., Lessov-Schlaggar C.N., Jain N., Cheng W.W.L., Komen H., Yee B., Bolzenius J.D., Janski A., Gibbons R., Zorumski C.F., Conway C.R. (2021). A phase 2 trial of inhaled nitrous oxide for treatment-resistant major depression.. Sci. Transl. Med..

[68] Yan D., Liu B., Wei X., Ou W., Liao M., Ji S., Peng Y., Liu J., Wu S., Wang M., Ju Y., Zhang L., Li Z., Li L., Zhang Y. (2022). Efficacy and safety of nitrous oxide for patients with treatment-resistant depression, a randomized controlled trial.. Psychiatry Res..

[69] Guimarães M.C., Guimarães T.M., Hallak J.E., Abrão J., Machado-de-Sousa J.P. (2021). Nitrous oxide as an adjunctive therapy in major depressive disorder: A randomized controlled double-blind pilot trial.. Br. J. Psychiatry.

[70] Liu W., Li Q., Ye B., Cao H., Shen F., Xu Z., Du W., Guo F., Liu J., Li T., Zhang B., Liu Z. (2020). Repeated nitrous oxide exposure exerts antidepressant-like effects through neuronal nitric oxide synthase activation in the medial prefrontal cortex.. Front. Psychiatry.

[71] Brown P.L., Zanos P., Wang L., Elmer G.I., Gould T.D., Shepard P.D. (2018). Isoflurane but not halothane prevents and reverses helpless behavior: A role for EEG burst suppression?. Int. J. Neuropsychopharmacol..

[72] Theilmann W., Rosenholm M., Hampel P., Löscher W., Rantamäki T. (2020). Lack of antidepressant effects of burst-suppressing isoflurane anesthesia in adult male Wistar outbred rats subjected to chronic mild stress.. PLoS One.

[73] Zhang S.S., Tian Y.H., Jin S.J., Wang W.C., Zhao J.X., Si X.M., Zhang L., Xu H., Jin J.Y. (2019). Isoflurane produces antidepressant effects inducing BDNF-TrkB signaling in CUMS mice.. Psychopharmacology.

[74] Antila H., Ryazantseva M., Popova D., Sipilä P., Guirado R., Kohtala S., Yalcin I., Lindholm J., Vesa L., Sato V., Cordeira J., Autio H., Kislin M., Rios M., Joca S., Casarotto P., Khiroug L., Lauri S., Taira T., Castrén E., Rantamäki T. (2017). Isoflurane produces antidepressant effects and induces TrkB signaling in rodents.. Sci. Rep..

[75] Jelen L.A., Stone J.M., Young A.H., Mehta M.A. (2022). The opioid system in depression.. Neurosci. Biobehav. Rev..

[76] Peciña M., Karp J.F., Mathew S., Todtenkopf M.S., Ehrich E.W., Zubieta J.K. (2019). Endogenous opioid system dysregulation in depression: Implications for new therapeutic approaches.. Mol. Psychiatry.

[77] Nummenmaa L., Karjalainen T., Isojärvi J., Kantonen T., Tuisku J., Kaasinen V., Joutsa J., Nuutila P., Kalliokoski K., Hirvonen J., Hietala J., Rinne J. (2020). Lowered endogenous mu-opioid receptor availability in subclinical depression and anxiety.. Neuropsychopharmacology.

[78] Besson A., Privat A.M., Fialip J., Eschalier A. (1996). Effects of morphine, naloxone and their interaction in the learned-helplessness paradigm in rats.. Psychopharmacology.

[79] Jiang C., DiLeone R.J., Pittenger C., Duman R.S. (2024). The endogenous opioid system in the medial prefrontal cortex mediates ketamine’s antidepressant-like actions.. Transl. Psychiatry.

[80] Zhang J., Lu Y., Jia M., Bai Y., Sun L., Dong Z., Tian W., Yin F., Wei S., Wang Y. (2023). Kappa opioid receptor in nucleus accumbens regulates depressive-like behaviors following prolonged morphine withdrawal in mice.. iScience.

[81] Boulos L.J., Nasseef M.T., McNicholas M., Mechling A., Harsan L.A., Darcq E., Ben Hamida S., Kieffer B.L. (2019). TouchScreen-based phenotyping: Altered stimulus/reward association and lower perseveration to gain a reward in mu opioid receptor knockout mice.. Sci. Rep..

[82] Karp J.F., Butters M.A., Begley A.E., Miller M.D., Lenze E.J., Blumberger D.M., Mulsant B.H., Reynolds C.F. (2014). Safety, tolerability, and clinical effect of low-dose buprenorphine for treatment-resistant depression in midlife and older adults.. J. Clin. Psychiatry.

[83] Paquelet G.E., Carrión K., Lacefield C.O., Zhou P., Hen R., Miller B.R. (2022). Single-cell activity and network properties of dorsal raphe nucleus serotonin neurons during emotionally salient behaviors.. Neuron.

[84] Welsch L., Colantonio E., Frison M., Johnson D.A., McClain S.P., Mathis V., Banghart M.R., Ben Hamida S., Darcq E., Kieffer B.L. (2023). Mu opioid receptors-expressing neurons in the dorsal raphe nucleus are involved in reward processing and affective behaviors.. Biol. Psychiatry.

[85] Estave P.M., Spodnick M.B., Karkhanis A.N. (2020). KOR control over addiction processing: An exploration of the mesolimbic dopamine pathway.. Handb. Exp. Pharmacol..

[86] He Y., Piao Z.S., Jia Y., Wu H.Q., Wang X.Q., Yu W.F., Wu F.X. (2025). Dezocine modulates the reinstatement of conditioned place preference in morphine-dependent rats *via* the dopamine reward circuitry.. Front. Neurosci..

[87] Negus S.S., St Onge C.M., Lee Y.K., Li M., Rice K.C., Zhang Y. (2024). Effects of selective and mixed-action kappa and delta opioid receptor agonists on pain-related behavioral depression in mice.. Molecules.

[88] Jacobson M.L., Wulf H.A., Browne C.A., Lucki I. (2020). The kappa opioid receptor antagonist aticaprant reverses behavioral effects from unpredictable chronic mild stress in male mice.. Psychopharmacology.

[89] Schmidt M.E., Kezic I., Popova V., Melkote R., Van Der Ark P., Pemberton D.J., Mareels G., Canuso C.M., Fava M., Drevets W.C. (2024). Efficacy and safety of aticaprant, a kappa receptor antagonist, adjunctive to oral SSRI/SNRI antidepressant in major depressive disorder: Results of a phase 2 randomized, double-blind, placebo-controlled study.. Neuropsychopharmacology.

[90] Browne C.A., Lucki I. (2019). Targeting opioid dysregulation in depression for the development of novel therapeutics.. Pharmacol. Ther..

[91] Olson K.M., Hillhouse T.M., Burgess G.E., West J.L., Hallahan J.E., Dripps I.J., Ladetto A.G., Rice K.C., Jutkiewicz E.M., Traynor J.R. (2023). Delta opioid receptor-mediated antidepressant-like effects of diprenorphine in mice.. J. Pharmacol. Exp. Ther..

[92] Saitoh A., Sugiyama A., Nemoto T., Fujii H., Wada K., Oka J.I., Nagase H., Yamada M. (2011). The novel δ opioid receptor agonist KNT-127 produces antidepressant-like and antinociceptive effects in mice without producing convulsions.. Behav. Brain Res..

[93] Yoshioka T., Yamada D., Segi-Nishida E., Nagase H., Saitoh A. (2023). KNT-127, a selective delta opioid receptor agonist, shows beneficial effects in the hippocampal dentate gyrus of a chronic vicarious social defeat stress mouse model.. Neuropharmacology.

[94] Torregrossa M.M., Jutkiewicz E.M., Mosberg H.I., Balboni G., Watson S.J., Woods J.H. (2006). Peptidic delta opioid receptor agonists produce antidepressant-like effects in the forced swim test and regulate BDNF mRNA expression in rats.. Brain Res..

[95] Weinerman J., Vazquez A., Schurhoff N., Shatz C., Goldenberg B., Constantinescu D., Hernandez G.M. (2023). The impacts of anxiety and depression on outcomes in orthopaedic trauma surgery: A narrative review.. Ann. Med. Surg..

[96] Scherrer J.F., Salas J., Copeland L.A., Stock E.M., Ahmedani B.K., Sullivan M.D., Burroughs T., Schneider F.D., Bucholz K.K., Lustman P.J. (2016). Prescription opioid duration, dose, and increased risk of depression in 3 large patient populations.. Ann. Fam. Med..

[97] Rosoff D.B., Smith G.D., Lohoff F.W. (2021). Prescription opioid use and risk for major depressive disorder and anxiety and stress-related disorders: A multivariable mendelian randomization analysis.. JAMA Psychiatry.

[98] Kaiya H. (2024). Anxious-depressive attack and rejection sensitivity-Toward a new approach to treatment-resistant depression.. Neuropsychopharmacol. Rep..

[99] Wu Z., Zhao P., Peng J., Fang L., Ding J., Yan G., Wang Y., Zhu J., Wang D., Li Y., Chen Z., Zhang Q., Deng Q., Duan G., Zuo Z., Li H. (2021). A patient-controlled intravenous analgesia with tramadol ameliorates postpartum depression in high-risk woman after cesarean section: A randomized controlled trial.. Front. Med..

[100] Ostadhadi S., Norouzi-Javidan A., Chamanara M., Akbarian R., Imran-Khan M., Ghasemi M., Dehpour A.R. (2017). Involvement of NMDA receptors in the antidepressant-like effect of tramadol in the mouse forced swimming test.. Brain Res. Bull.

[101] Arakawa R., Takano A., Halldin C. (2019). Serotonin and norepinephrine transporter occupancy of tramadol in nonhuman primate using positron emission tomography.. Int. J. Neuropsychopharmacol..

[102] Caspani O., Reitz M.C., Ceci A., Kremer A., Treede R.D. (2014). Tramadol reduces anxiety-related and depression-associated behaviors presumably induced by pain in the chronic constriction injury model of neuropathic pain in rats.. Pharmacol. Biochem. Behav..

[103] Roussin A., Soeiro T., Fouque C., Jouanjus E., Frauger E., Fouilhé N., Mallaret M., Micallef J., Lapeyre-Mestre M. (2022). Increase of high-risk tramadol use and harmful consequences in France from 2013 to 2018: Evidence from the triangulation of addictovigilance data.. Br. J. Clin. Pharmacol..

[104] Arve K. (2023). “You get stuck in it”: Young people’s accounts of attempting to quit non-medical tramadol use.. Nord Alkohol- Narkotikatidskrift.

